# Resistant Starch and Microbiota-Derived Secondary Metabolites: A Focus on Postbiotic Pathways in Gut Health and Irritable Bowel Syndrome

**DOI:** 10.3390/ijms26167753

**Published:** 2025-08-11

**Authors:** Eniko Kovacs, Katalin Szabo, Rodica-Anita Varvara, Alina Uifãlean, Angela Cozma, Romana Vulturar, Adela Viviana Sitar-Taut, Rosita Gabbianelli, Mari C. W. Myhrstad, Vibeke H. Telle-Hansen, Olga Hilda Orãșan, Adriana Fodor, Ramona Suharoschi, Simona-Codruţa Hegheș

**Affiliations:** 1 Department of Food Science, University of Agricultural Science and Veterinary Medicine, 3-5 Calea Mănăștur, 400372 Cluj-Napoca, Romania; keniko70@gmail.com (E.K.); rodica-anita.varvara@student.usamvcluj.ro (R.-A.V.); 2 Molecular Nutrition and Proteomics Laboratory, CDS3, Institute of Life Sciences, University of Agricultural Sciences and Veterinary Medicine, 400372 Cluj-Napoca, Romania; 3 Technological Transfer Center COMPAC, University of Agricultural Sciences and Veterinary Medicine, 400372 Cluj-Napoca, Romania; katalin.szabo@usamvcluj.ro; 4 Department of Drug Analysis, “Iuliu Hațieganu” University of Medicine and Pharmacy, 6 Louis Pasteur Street, 400349 Cluj-Napoca, Romania; alina.uifalean@umfcluj.ro (A.U.); cmaier@umfcluj.ro (S.-C.H.); 5 Department 4th of Internal Medicine, Faculty of Medicine, Iuliu Hațieganu University of Medicine and Pharmacy, 400012 Cluj-Napoca, Romania; angelacozma@yahoo.com (A.C.); adelasitar@yahoo.com (A.V.S.-T.); olgaorasan@gmail.com (O.H.O.); 6 Department of Molecular Sciences, “Iuliu Hațieganu” University of Medicine and Pharmacy, 6 Louis Pasteur St., 400349 Cluj-Napoca, Romania; romanavulturar@gmail.com; 7 Unit of Molecular Biology and Nutrigenomics, University of Camerino, via Gentile III da Varano, 62032 Camerino, Italy; rosita.gabbianelli@unicam.it; 8 Department of Nursing and Health Promotion, Faculty of Health Sciences, Oslo Metropolitan University, Postbox 4, St. Olavs Plass, 0130 Oslo, Norway; mmyhrsta@oslomet.no (M.C.W.M.); vtelle@oslomet.no (V.H.T.-H.); 9 Clinical Center of Diabetes, Nutrition and Metabolic Diseases, “Iuliu Hatieganu” University of Medicine and Pharmacy, 2-4 Clinicilor St., 400012 Cluj-Napoca, Romania; adifodor@yahoo.com

**Keywords:** resistant starch (RS), postbiotics, gut microbiota, personalized nutrition, irritable bowel syndrome (IBS), short-chain fatty acids (SCFAs), delivery system, gut microbiome

## Abstract

Resistant starch (RS) is emerging as a multifunctional dietary component and delivery platform for microbiota-accessible carbohydrates. Upon fermentation by gut microbiota, particularly in the colon, RS generates a wide spectrum of postbiotic compounds—including short-chain fatty acids (SCFAs), indoles, bile acid derivatives, and neuroactive amines such as GABA and serotonin precursors. These metabolites modulate gut–brain signaling, immune responses, and intestinal barrier integrity, which are critical pathways in the pathophysiology of irritable bowel syndrome (IBS). This review synthesizes current knowledge on RS structure, classification, and fermentation dynamics, with a special focus on RS3 due to its practical dietary relevance and strong microbiota-modulatory effects. We highlight emerging evidence from clinical studies supporting RS-mediated improvements in IBS symptoms, microbial diversity, and inflammation. Importantly, RS acts as a smart colonic delivery system by escaping enzymatic digestion in the small intestine and reaching the colon intact, where it serves as a targeted substrate for microbial fermentation into bioactive metabolites. This host–microbiota interplay underpins the development of personalized, microbiome-informed nutrition interventions tailored to specific IBS subtypes. Future directions include omics-based stratification, optimized RS formulations, and predictive algorithms for individualized responses. This review aims to clarify the mechanistic links between RS fermentation and postbiotic production, highlighting its therapeutic potential in IBS management.

## 1. Introduction

Irritable bowel syndrome (IBS) is a common functional gastrointestinal disorder with a very broad estimated prevalence (1.1–45.0%) due to a large methodological heterogeneity [1,2]. IBS exhibits a higher incidence in women [3]. Characterized by multifactorial etiology, IBS poses diagnostic and therapeutic challenges due to the absence of pathognomonic structural changes. Resistant starch (RS) is emerging as a multifunctional delivery system for bioactive metabolite generation through targeted microbiota fermentation. By promoting the site-specific production of postbiotics such as SCFAs, indoles, and neuroactive amines, RS influences gut–brain signaling, intestinal barrier function, and immune modulation—key pathways in irritable bowel syndrome (IBS) [4].

IBS manifests in four subtypes based on stool patterns: diarrhea-dominant (IBS-D), constipation-dominant (IBS-C), mixed diarrhea and constipation-dominant (IBS-M), and uncategorized (IBS-U), with classification relying on the Bristol Scale [2,5]. The etiopathogenesis of IBS encompasses diverse factors, including genetics, dietary habits, gut microbiota alterations, gastrointestinal motility, gut endocrine cells, gut–brain axis modulation, psychosocial factors, low grade gastrointestinal inflammation, and food intolerance. Notably, alterations in gut microbiota, scrutinized through advanced techniques like 16S rRNA sequencing, reveal dysbiosis at both the colon mucosa and lumen levels in IBS patients [6,7]. Distinct microbiota compositions further correlate with specific disease symptomatology [8].

Despite lacking life-threatening implications, IBS significantly impairs patients’ quality of life (QoL), fostering food intolerances, physical inactivity, limited social engagement, and comorbidities such as fibromyalgia, chronic fatigue syndrome, chronic pelvic pain, psycho-emotional disorders, gastrointestinal disorders, and gastroesophageal reflux [9]. Treatment modalities, encompassing pharmacological and non-pharmacological approaches, encounter limitations due to disease heterogeneity, symptom fluctuations, and lifestyle factors. While fecal microbiota transplantation holds promise, further research and protocol development are imperative. In the realm of non-pharmacological interventions, diet assumes a pivotal role, with prebiotic dietary fibers constituting a fundamental aspect of IBS management. RS manipulation of the gut microbiota emerges as a promising avenue, demonstrating significant results and ongoing developments [10]. Future research should emphasize the formulation of effective treatment strategies addressing the root cause of IBS, aiming to enhance QoL for affected individuals.

**Figure 1:**
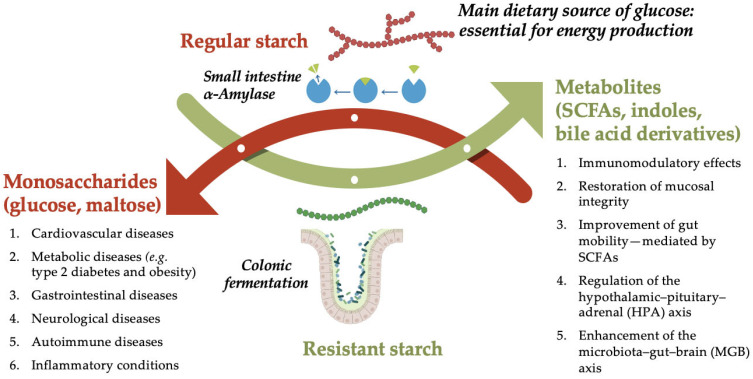
Comparison of regular and resistant starch in the human GI tract: Digestion vs. Fermentation. Schematic representation of the differential metabolic fates and health impacts of regular starch versus resistant starch. Regular starch is enzymatically hydrolyzed by α-amylase in the small intestine (indicated by the blue arrow), producing monosaccharides such as glucose and maltose, which are rapidly absorbed and utilized for energy production. However, excessive intake of rapidly digestible starches is associated with increased risk of chronic diseases, including cardiovascular, metabolic (e.g., type 2 diabetes, obesity), gastrointestinal, neurological, autoimmune, and other inflammatory conditions. In contrast, resistant starch bypasses digestion in the small intestine and reaches the colon intact, where it undergoes microbial fermentation. This process generates beneficial metabolites such as short-chain fatty acids (SCFAs), indoles, and bile acid derivatives, which exert multiple health-promoting effects. These include immunomodulatory activity, restoration of mucosal integrity, improved gut motility (mediated by SCFAs), regulation of the hypothalamic–pituitary–adrenal (HPA) axis, and enhancement of the microbiota–gut–brain (MGB) axis. Created in PresentationGO. Heghes S.C. (2025) https://PresentationGO.com (accessed on 8 July 2025).

Starch is a polymeric carbohydrate composed of glucose units linked by glycosidic bonds and represents the primary source of dietary carbohydrates, contributing approximately 45–65% of the total daily energy intake in humans. Based on its digestibility, starch is classified into three major types: rapidly digestible starch (RDS), slowly digestible starch (SDS), and resistant starch (RS) [11]. RDS is quickly hydrolyzed in the small intestine by α-amylase, leading to a rapid rise in postprandial blood glucose and insulin levels; it is commonly found in processed foods such as white bread or instant cereals. SDS is digested more gradually, resulting in a slower and sustained glucose release; it is present in legumes, minimally processed whole grains, and pasta cooked al dente. RS, in contrast, resists enzymatic digestion in the small intestine and reaches the colon intact, where it undergoes microbial fermentation, generating bioactive postbiotic metabolites. Sources of RS include cooked and cooled potatoes, rice, and pasta (RS3), as well as green bananas and high-amylose maize (RS2) [12]. The excessive intake of rapidly digestible starches has been associated with adverse effects on metabolic health and intestinal barrier function, including dysbiosis, increased intestinal permeability, and inflammation [12,13]. In contrast, slowly digestible and resistant starches exhibit distinct physiological effects [14]. Resistant starch, in particular, escapes enzymatic digestion in the small intestine and reaches the colon intact, where it undergoes microbial fermentation and contributes to metabolic and gut health. Figure 1 illustrates the health implications resulting from the indigestibility of RS.

The principal impact of resistant starch (RS) within the colon lies in its role as a substrate for microbiota, fostering the proliferation of beneficial bacteria. In recent years, this microbial fermentation of RS has gained renewed interest not only for its production of SCFAs, but also for the formation of a broader spectrum of biologically active compounds, that are collectively referred to as postbiotics. These include bile acid derivatives, tryptophan metabolites (such as indole-3-propionic acid and indole-3-acetic acid), and neuroactive amines like GABA. Their role in gut–brain communication, immune modulation, and metabolic regulation suggests potential systemic benefits that extend beyond the colon, especially in the context of IBS [15,16,17].

Due to their significant implications for irritable bowel syndrome (IBS), SCFAs emerge as a focal point of considerable interest in research, thus constituting the primary emphasis of this article.

RS, initially introduced by Englyst (1996) [18], refers to a fraction of dietary starch that escapes enzymatic digestion in the small intestine due to its distinct structural and physicochemical characteristics [19]. This property enables RS to reach the colon intact, where it undergoes microbial fermentation with important physiological effects. This review aims to provide a comprehensive overview of the interactions between resistant starch and the gut microbiota, with focus on fermentation-derived postbiotic compounds—short-chain fatty acids, indoles, and bile acid derivatives—and their relevance to irritable bowel syndrome (IBS). Additionally, it highlights current knowledge gaps and evaluates the potential of personalized nutritional strategies targeting these pathways.

## 2. Resistant Starch (RS)

### 2.1. Structure of RS

Starch is an intricate polysaccharide with the general chemical formula (C_6_H_10_O_5_)_n_. It consists of two main glucose polymers: amylose and amylopectin, which critically influence its physicochemical characteristics [20]. Amylose has a predominantly linear structure with α(1→4) glycosidic bonds, whereas amylopectin is highly branched, containing both α(1→4) and α(1→6) linkages [21]. The amylose-to-amylopectin ratio plays a crucial role in determining starch properties, including its crystallinity, grain architecture, digestibility, and glycemic index. Starch gels rich in amylopectin tend to be soft and exhibit rapid gelation, while gels with higher amylose content are firmer and more rigid [21,22]. Linear amylose contributes significantly to the formation of dense, retrograded structures, playing a major role in the generation of RS3. In contrast, the highly branched structure of amylopectin limits this retrogradation capacity and tends to reduce the starch’s resistance to digestion [23,24].

Due to its thermal stability, starch is widely used in the food industry. Its functional and nutritional properties can be enhanced through a range of physical (thermal, mechanical, radio-thermal) and chemical (oxidation, hydrolysis, esterification, etherification) modification techniques [25]. Scientifically, three distinct crystal structures have been identified in starch molecules. The A-type crystalline structure, prevalent in cereals, is associated with starch rich in amylopectin. It is characterized by a densely packed orthorhombic arrangement of double helices with limited interstitial water that facilitates faster gelatinization and renders the starch more susceptible to enzymatic hydrolysis [26,27]. In contrast, the B-type crystalline structure, predominantly found in tubers and high-amylose starches, exhibits a more relaxed hexagonal structure and a higher degree of hydration. This open conformation enhances structural rigidity and reduces enzymatic accessibility, contributing to its role in resistant starch [21,22]. The C-type structure represents a composite of both A- and B-type motifs and is frequently encountered in leguminous starches. Its heterogeneous crystalline domains confer intermediate digestibility, contingent on the proportion and distribution of A- and B-type regions within the granule [21,22,26,28].

Regarding the application of nutritional therapy in the management of IBS, the efficacy of resistant starch (RS) is closely related to its enzymatic digestibility. Starch is generally classified into three categories based on digestion rate: rapidly digestible starch (RDS), slowly digestible starch (SDS), and resistant starch (RS) [29,30]. RS resists digestion in the small intestine and reaches the colon, where it undergoes fermentation by gut microbiota, producing beneficial short-chain fatty acids (SCFAs). This distinctive property renders it of considerable medical interest.

### 2.2. Types of RS

According to their origin and molecular structure, resistant starches are divided into five major types (RS1–RS5), as summarized in Table 1.

Type 1 (RS1)—Physically Inaccessible Starch: Naturally found in whole grains, seeds, and legumes, or dense food matrices such as partially extracted durum wheat, where starch granules are physically trapped within intact cell walls or fibrous matrices [31,34]. This structural barrier prevents enzymatic access, making RS1 resistant to digestion in the small intestine. The same resistance can also be achieved through technological processes such as encapsulation. Diets rich in RS1 have been associated with improved glycemic control, reduced postprandial insulin response, and beneficial effects on the gut microbiota through fermentation in the colon, potentially contributing to metabolic and cardiovascular health [29,34].

Type 2 (RS2): Present in various foods such as plantains or unripe bananas, raw potatoes, ginkgo biloba root, and high-amylose maize starch. Its resistance to digestion is due to a compact, crystalline granular structure that limits enzymatic access. Notably, type B and C crystalline structures exhibit heightened resistance, as enzymes can only create minor lesions on the granule surface [22,31]. Although RS2 loses its resistance upon conventional cooking, mild thermal or enzymatic treatments can help preserve its granular integrity [23]. Its inclusion in raw or specially processed products has been associated with an improved glycemic response, enhanced satiety, and support for colonic fermentation processes that yield beneficial short-chain fatty acids [23,24].

Type 3 (RS3) Retrograded Starch: RS3 forms during the process of gelatinization and the subsequent retrogradation of starch, typically in cooked and cooled starchy foods. Upon heating, the crystalline regions melt, and upon cooling, new crystalline structures (mainly A- and B-types) form, making the starch less digestible and more fermentable. This reversible process depends on temperature, storage duration, and the botanical source of starch [32,35,36]. Further details on RS3 formation, stability, and microbiota modulation are discussed in Section Focus on RS3.

Type 4 (RS4) is a chemically modified form of starch. It is obtained through processes such as esterification, etherification, or cross-linking [32,35,36]. These structural alterations increase its resistance to enzymatic digestion in the small intestine, rendering the starch resistant to enzymatic digestion in the small intestine, yet still fermentable by colonic bacteria. Cross-linking involves the chemical bonding of distinct starch molecules, enhancing structural rigidity and reducing digestibility. Octenyl succinic groups are incorporated into starch molecules via esterification to enhance their water solubility and emulsification properties, whereas acylation introduces fatty acid-like groups, enhancing thermal stability and enzymatic resistance. In essence, RS4 serves as a valuable dietary fiber source that contributes to digestive health and overall well-being [37]. Since RS4 is obtained through chemical reactions such as esterification or cross-linking, its use in food products is strictly regulated and requires approval in many jurisdictions, including the European Union and the United States [36].

RS type 5 (RS5) results from the complexation between starch, particularly amylose, and lipids such as fatty acids or fatty alcohols, forming thermally stable, enzyme-resistant V-type helical complexes [32,33]. The stability of RS5 is significantly influenced by the type of lipid: saturated fatty acids form more stable complexes, whereas unsaturated fatty acids yield less resistant structures [38,39]. Unlike most starches that lose resistance upon cooking, RS5 maintains or rapidly regains its resistant structure due to the instantaneous reformation of the complex during cooling. Its heat stability and potential to modulate lipid absorption and glycemic response make it a topic of emerging interest in functional nutrition.

#### Focus on RS3

This study places particular emphasis on RS3, the retrograded starch formed through the cooking and subsequent cooling of starchy foods. RS3 differentiates itself by its practical applicability in daily diets and by its strong microbiota modulation effects [40]. Unlike other forms of resistant starch described before, that are either native resistant or rendered so through chemical modulation, RS3 results from structural transformations that occur in starch molecules upon heating and subsequent cooling. The process begins with gelatinization, during which heat, and water disrupt the crystalline structures of the starch granules. This leads to the swelling of the granules and alteration of the crystalline architecture, particularly of amylopectin, resulting in a gel or paste that is initially more digestible [35,41,42]. During cooling, gelatinized starch undergoes retrogradation, a process involving the reassociation of linear amylose chains and amylopectin segments into more ordered crystalline configurations. The newly formed crystals typically adopt A-type or B-type arrangements, depending on the starch source and cooling conditions. These retrograded regions are less susceptible to enzymatic hydrolysis and thus constitute the resistant fraction [35,36]. The final phase of retrogradation, known as maturation, involves the structural reorganization and densification of preformed crystalline regions. This step enhances the thermodynamic stability and enzymatic resistance of RS3, often increasing its crystallinity during extended storage, particularly under ambient conditions [36]. This progressive transition is characterized by an advanced organization and the potential formation of type B crystalline polymorphs, involving the phenomenon of syneresis or the expulsion of water from the starch gel matrix [41].

**Figure 2:**
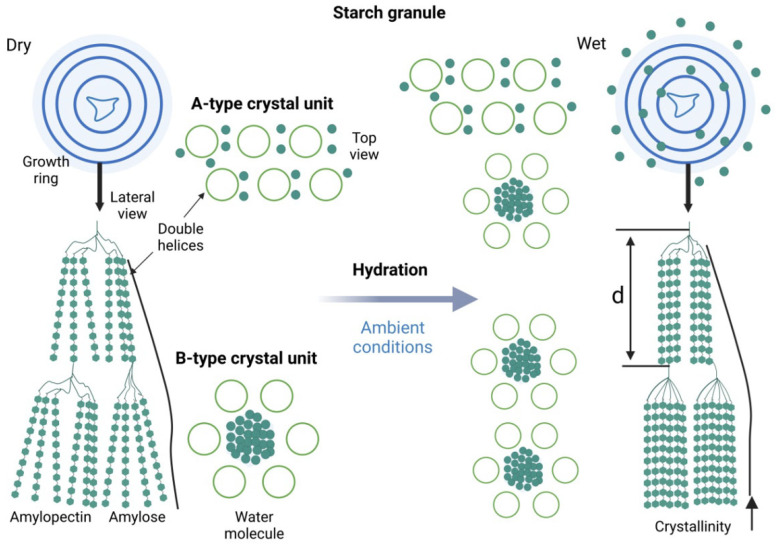
Structural organization and hydration-induced transformation of starch granules, highlighting A- and B-type crystalline units and their relevance to RS3 formation. Created in Biorender. Varvara R. (2025) https://app.biorender.com/illustrations/68947947011fb7309a7391c8 (accessed on 8 July 2025).

These structural transitions are illustrated in Figure 2, which depicts the conversion of starch granules from a hydrated amorphous state into crystalline domains under ambient conditions.

The formation and stability of RS3 depends on a series of interrelated factors, including temperature, water content, starch composition, and storage time. Cooling at 4 °C has been shown to accelerate retrogradation, especially in starches with high amylose contents. While this will promote early nucleation and double-helix formation, Li et al. (2024) [43] demonstrated that a slow nucleation process could also be initiated at 25 °C, leading to increased thermodynamic stability and reduced enzymatic susceptibility after the propagation and maturation phases. However, prolonged refrigeration beyond 48 h does not seem to have a major impact on the subsequent increase in strength as the organization of the crystal lattice tends to decrease. In contrast, prolonged storage at an ambient temperature (~25 °C) facilitates the maturation phase, during which the crystalline regions reorganize into more thermodynamically stable structures, increasing the overall strength [35,42,44]. According to Gong et al. (2024) [36], optimal retrogradation was achieved when the water content was maintained between 30% and 50%, enabling chain mobility without excessive disruption of hydrogen bonding. Storage at 25 °C was found to promote continuous crystalline rearrangement, whereas lower or higher moisture levels impeded helix formation. Furthermore, repeated cooling–reheating cycles may further reinforce the crystalline architecture of RS3, leading to increased enzymatic resistance and SCFA production upon fermentation [43].

Due to its thermal stability and ability to be generated through repeated cooking and cooling, RS3 represents an effective and practical dietary fiber. Foods such as cooked and cooled potatoes, rice, pasta, and legumes are naturally enriched in this starch fraction, making RS3 both technologically and nutritionally relevant. Given these characteristics, RS3 stands out not only for its structural and physicochemical properties but also for its accessibility in common diets, facilitating its integration into microbiota-targeted nutritional interventions—particularly in the management of IBS.

### 2.3. Occurrence and Modulation of Resistant Starch in Foods

The presence of resistant starch (RS) in food products is shaped by both botanical origin and processing conditions. Its naturally occurring levels vary considerably depending on the source material, cooking method, and storage regime, ranging from negligible amounts to up to 20% of the total product weight [45]. Thermal treatment is a key determinant: while heating (gelatinization) typically disrupts RS2 structures, subsequent cooling facilitates the formation of RS3 through retrogradation. This mechanism is especially relevant in foods like rice, pasta, and potatoes, as well as in less commonly explored sources such as green bananas [46], mung beans, and sweet potatoes [20]. Storage, particularly at low temperatures, enhances RS levels further due to amylopectin recrystallization. For example, cooked potatoes stored at 0 °C exhibit a significantly greater RS3 yield than those kept at room temperature. Repeated cooling–heating cycles may further consolidate crystalline domains, thereby increasing the resistant fraction.

In baked foods, RS content tends to rise as a result of partial gelatinization followed by retrogradation during cooling. The addition of high-amylose starch or lactic acid to dough formulations has been shown to further elevate RS levels. In confectionery, similar increases may be achieved by optimizing formulation and temperature conditions. Cooking methods also play a pivotal role in RS formation. For instance, fried potatoes typically retain more RS than boiled ones, due to reduced moisture and the preservation of crystalline zones. In rice, steaming followed by refrigeration is more conducive to RS3 accumulation than boiling and immediate consumption [36,47].

Beyond domestic practices, various industrial and lab-scale strategies have been implemented to enhance starch retrogradation and boost RS3 formation. These include physical, enzymatic, and chemical modifications that reorganize starch granule structure, improve gel strength, and increase resistance to enzymatic degradation.

Among physical treatments, heat-moisture treatment (HMT) at intermediate moisture (23–30%) and elevated temperatures (85–130 °C) has been shown to reduce pasting viscosity while improving gel strength and thermal stability in starches from potato and buckwheat [36,48]. Dry heat treatment (DHT) applied to wheat or rice starch, enhances rigidity and supports the formation of stable crystalline structures [36,47,49]. Microwave (MT) and ultrasound treatments (UT), when carefully controlled, enhance viscoelastic properties and facilitate compact gel formation, as reported for potato, quinoa, and lotus starches [20,36,50]. However, overtreatment may compromise the structural integrity of the network.

Enzymatic approaches, such as pullulanase or α-amylase application, contribute by cleaving α(1→6) linkages in amylopectin, allowing for linear alignment and more efficient double-helix formation during retrogradation [51,52,53]. Enzyme-treated starches, when combined with hydrocolloids like xanthan gum or sodium alginate, result in cohesive gels with enhanced rheological behavior [54].

Chemical cross-linking using agents such as lactic or citric acid, or phosphorylating reagents like STMP (Sodium Trimetaphosphate)/STPP (Sodium Tripolyphosphate), induces covalent linkages that stabilize starch matrices. These treatments improve freeze–thaw resistance and prevent granule disintegration during gelatinization, supporting the retention of crystalline domains and higher RS yields [55,56]. Also, emerging approaches include the development of RS3-based starch nanoparticles, which exhibit high stability and potential for use as functional carriers in nutrient delivery systems [20].

Altogether, these strategies provide robust tools for increasing RS3 content in food systems, enabling the development of functional products that support colonic health, modulate glycemic responses, and contribute to the dietary management of metabolic diseases.

In addition to naturally occurring and processing-induced RS, commercial preparations of resistant starch have become increasingly available, and they are widely used in functional food formulations. Notable examples include Hi-maize^®^ (Ingredion, Westchester, IL, USA) [57], derived from high-amylose maize and primarily classified as RS2, and Novelose^®^ starches (Ingredion), which include RS2, RS3, and RS4 forms depending on the modification process [58]. Another example is Solnul™ (MGP Ingredients, Atchison, KS, USA), a RS4-type resistant wheat starch obtained through cross-linking with food-grade chemicals [59]. These products are engineered to have predictable physicochemical properties, including a high RS content (often >60%), improved thermal stability, low water absorption, and a neutral taste, making them suitable for incorporation into bakery items, snacks, nutrition bars, and beverages. Their consistent composition allows manufacturers to meet dietary fiber labeling requirements due to the possibility of standardization [57,58,59].

### 2.4. Microbial Fermentation of RS and SCFAs Production

Unlike rapidly digestible starch (RDS) and slowly digestible starch (SDS), resistant starch (RS) escapes hydrolysis by pancreatic α-amylase in the small intestine, reaching the colon intact. SCFAs represent the principal metabolites resulting from the microbial fermentation of indigestible carbohydrates, including resistant starch. The most prevalent SCFAs are acetate (C2), propionate (C3), and butyrate (C4). The quantitative and qualitative SCFA profile within the colon is shaped by multiple factors, including the type of fermentable substrate (e.g., resistant starch vs. soluble fibers), the colonic transit time, and the composition of the resident microbiota [60,61,62,63].

Acetate is synthesized by a broad spectrum of bacterial genera, including *Bacteroides* spp., *Akkermansia muciniphila*, *Clostridium* spp., and *Bifidobacterium* spp., primarily via the acetyl-CoA pathway. It serves as a key substrate for peripheral lipogenesis and cholesterol biosynthesis [64]. In addition to its systemic metabolic roles, acetate contributes to mucosal defense and anti-inflammatory signaling through the activation of G-protein-coupled receptor 43 (GPR43) [65,66].

Propionate is produced via three main microbial pathways: (i) the succinate pathway (predominant in *Bacteroides* spp. and *Veillonella* spp.), (ii) the acrylate pathway (employed by *Clostridium propionicum* and *Megasphaera elsdenii*), and (iii) the propanediol pathway (active in *Roseburia inulinivorans* and *Blautia* spp.) [63,64]. These metabolic routes not only define the specific taxa capable of propionate synthesis but also influence its concentration and availability in different gut segments. The dominance of the succinate pathway, particularly among *Bacteroides* species, links propionate production closely with dietary fiber and RS fermentation profiles [67]. Propionate is predominantly metabolized in the liver, where it participates in gluconeogenesis and modulates lipid metabolism. It also exerts regulatory effects on satiety and immune responses via GPR41-mediated pathways [68].

Butyrate, although less abundant, is particularly significant due to its multifaceted role in maintaining colonic health. It serves as the primary energy source for colonocytes, supports mucosal barrier integrity, and exhibits marked anti-inflammatory activity through inhibition of histone deacetylases (HDAC) and activation of GPR109A [64]. It thus suppresses the synthesis of pro-inflammatory cytokines including interleukin-6 (IL-6) and tumor necrosis factor-alpha (TNF-α) and promotes the expression of IL-10, an anti-inflammatory cytokine with protective roles in intestinal homeostasis [69]. These effects are partially mediated by inhibition of the NF-κB signaling pathway, as well as by the upregulation of cytokine signaling suppressor proteins such as SOCS3 [70,71]. In addition to its immunological impact, butyrate supports epithelial barrier integrity by serving as a primary energy substrate for colonocytes and stimulating the release of glucagon-like peptide-2 (GLP-2) from enteroendocrine L-cells. This in turn enhances the expression of tight junction proteins and the transcription of the MUC2 gene, thereby promoting mucin production and epithelial regeneration [72].

The microbial synthesis of butyrate from resistant starch involves a well-coordinated enzymatic and structural interaction with the substrate. Starch degradation is mediated by a set of specialized enzymes, including α-amylase, type I pullulanase, and amylopullulanases, each targeting specific glycosidic linkages within the starch molecule [52]. The fermentation process initiates with the binding of the starch molecule to bacterial structures, with at least two recognized points of connection. The first involves the cellulosome present in *Ruminococcus flavefaciens*, while the second involves the outer membrane protein complex in *Bacteroides thetaiotaomicron*. Gram-positive bacteria establish connections through cell-associated α-amylase, identified in *Butyrivibrio fibrisolvens*, *Roseburia inulinviorans*, and *Roseburia intestinalis*, known for butyrate production. Barcenilla et al. (2000) used 16S rRNA sequencing to pinpoint bacteria central to butyrate production, including *Eubacterium rectale*, *Eubacterium ramulus*, *Roseburia cecicola*, and the *Clostridium leptum* group [73]. Advancements in metagenomics and transcriptomics sequencing have enabled the identification of key genes involved in butyrate synthesis, most notably butyrate kinase and butyryl-CoA:acetate-CoA transferase [74]. In their study, Louis et al. (2004) reported that the gene encoding butyrate kinase was present in only 4 of 38 bacterial strains analyzed, whereas the gene encoding butyryl-CoA:acetate-CoA transferase was present in all 38 strains [75,76]. These findings suggest that the latter is the dominant pathway for butyrate formation in the human colon. The presence of methanogenic Archaea, such as *Methanobrevibacter smithii*, in the gut microbiota may negatively influence butyrate production as their abundance has been inversely correlated with short-chain fatty acid concentrations. Recent data show substantial interethnic variations in *M. smithii* prevalence, ranging from 22–28% in Asian populations to 45–50% in Caucasians, suggesting that archaeal–bacterial interactions may partially explain population-level differences in SCFA profiles [77].

SCFA concentrations diminish progressively along the colon, from 70–140 mM in the proximal colon to 20–70 mM in the distal colon, corresponding to substrate depletion. Prolonged transit time can enhance protein breakdown, potentially increasing the SCFA pool. Additionally, an elevated intake of RS may modify the concentration of SCFAs in the colon and feces [62].

Emerging evidence also suggests a functional connection between short-chain fatty acids and the enteric nervous system (ENS). Butyrate and other SCFAs stimulate enteroendocrine signaling through the release of neuroactive peptides, including 5-hydroxytryptamine (5-HT), VIP, somatostatin, and cholecystokinin, which act via paracrine mechanisms to regulate intestinal motility. This crosstalk is further supported by the activity of monocarboxylate transporter 2 (MCT2) expressed in enteric neurons, which facilitates SCFA uptake and downstream neuromodulatory effects [78,79].

Taken together, these findings highlight the multidimensional non-pharmacological therapeutic relevance of an RS diet, not only as a source of a microbial metabolites but as key mediators of gut immune balance, epithelial repair, and neuroenteric signaling.

### 2.5. Other Microbial Metabolites Influenced by RS Fermentation

In addition to short-chain fatty acids directly produced by fermentation of RS by colonic microbiota, a wide array of microbial-derived metabolites with diverse biological activities can indirectly be modulated by RS intake. These compounds include indoles, derivatives of bile acids, biogenic amines, microbial neurotransmitters, and bioactive peptides, each contributing uniquely to host physiology and intestinal homeostasis [64,68].

Indole and its derivatives, produced through the bacterial metabolism of dietary tryptophan, exert significant effects on epithelial barrier integrity, immune modulation, and enteroendocrine signaling via activation of the aryl hydrocarbon receptor (AhR) [78]. Specific indole derivatives such as indole-3-propionic acid have been shown to protect against oxidative stress and neuroinflammation [64].

Secondary bile acids, including deoxycholic acid and lithocholic acid, result from the microbial transformation of primary bile acids through 7α-dehydroxylation [80]. These compounds can modulate lipid absorption, influence gut motility, and act as signaling molecules via the farnesoid X receptor (FXR) and G-protein-coupled bile acid receptor 1 (TGR5), with downstream effects on metabolism and inflammation [81].

Biogenic amines, such as histamine, tyramine, cadaverine, and putrescine, are formed through the decarboxylation of amino acids by specific microbial decarboxylases [82,83]. While low concentrations may exert signaling or regulatory functions, excessive accumulation can contribute to mucosal irritation, dysbiosis, or the exacerbation of IBS symptoms [68].

Microbial neurotransmitters represent a key aspect of the microbiota-gut–brain axis. Gamma-aminobutyric acid (GABA), serotonin (5-HT), and dopamine precursors are synthesized by various gut bacteria, including *Lactobacillus* spp. and *Bifidobacterium* spp. [78,84]. GABA is involved in intestinal motility and barrier regulation, while microbial serotonin production may influence both gastrointestinal and central nervous system functions [16]. Moreover, certain microbial strains can release anti-inflammatory peptides and small proteins that interact with host immune pathways, reducing the expression of pro-inflammatory cytokines and enhancing epithelial repair processes [15].

Additionally, the fermentation of RS can give rise to volatile organic compounds (VOCs), such as alcohols, ketones, aldehydes, and sulfur-containing molecules [64]. These compounds may modulate microbial crosstalk, contribute to gut olfactory signaling, or serve as potential biomarkers of microbial activity and dysbiosis [85]. In parallel, gaseous fermentation products, such as hydrogen, methane, and carbon dioxide are also released during RS metabolism [86]. These gases may influence gut physiology by altering luminal pressure, promoting distension, and modulating visceral sensitivity, especially in IBS patients [87,88].

Together, these microbial metabolites complement the actions of SCFAs and expand the functional relevance of RS fermentation. Their cumulative impact on intestinal health, immune modulation, metabolic regulation, and neurobehavioral signaling underscores the therapeutic potential of resistant starch as a modulator of host–microbiota interactions.

## 3. Irritable Bowel Syndrome (IBS)

The nomenclature “irritable colon” was originally introduced in 1929 to delineate a musculo-neuronal pathology of the colon marked by abdominal pain and disruptions in intestinal transit [89]. Brown, in 1947 expounded upon the concept of the “irritable bowel”, subsequently leading to the formal introduction of the term “irritable bowel syndrome” [90].

### 3.1. Diagnostic Criteria and Subtypes

**Figure 3:**
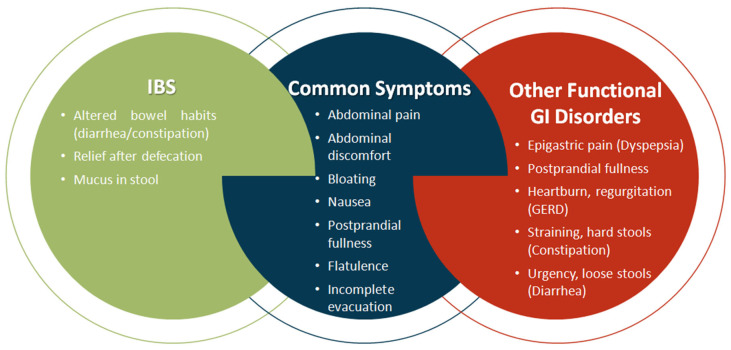
Symptom overlaps between IBS and Other Functional Gastrointestinal Disorders. Created in PresentationGO. Heghes S.C. (2025) https://PresentationGO.com (accessed on 8 July 2025).

IBS manifests as a condition typified by abdominal discomfort or pain, abdominal distention, flatulence, and disturbances in intestinal transit. Despite these hallmark features, the diagnostic process for IBS is intricate due to clinical presentations that may overlap with signs and symptoms indicative of other gastrointestinal disorders, including functional dyspepsia, chronic constipation, bloating, distension, and/or gastroesophageal reflux disease (GERD) [91,92]. The intricate intersection of IBS symptoms with those of alternative gastrointestinal maladies is elucidated in Figure 3.

Henceforth, the primary diagnostic undertaking involves the systematic exclusion of concomitant organic gastrointestinal pathologies, such as ulcerative colitis, Crohn’s disease, and colorectal cancer, predicated by both symptomatology and paraclinical investigations. While certain biomarkers—namely interleukin-1ß (IL-1ß), growth-related oncogene-a, brain-derived neurotrophic factor (BDNF), anti-*Saccharomyces cerevisiae* antibody (ASCA), anti-flagellin antibodies (anti-CBir1), anti-tissue transglutaminase (tTG), tumor necrosis factor (TNF), anti-neutrophil cytoplasmic antibody (ANCA), tissue inhibitor of metalloproteinase-1 (TIMP-1), and neutrophil gelatinase-associated lipocalin (NGAL)—may be considered in the diagnostic process, their utility is constrained by the absence of definitive serological and imaging alterations. Consequently, these markers are relegated to the status of adjunctive tools in the diagnostic arsenal [93,94].

The Manning criteria (1978) represents the inaugural diagnostic criterion for IBS. In accordance with these criteria, the primary symptoms encompass alterations in bowel movements coupled with features such as “abdominal pain, pain alleviation post-defecation, abdominal distension, passage of mucus, or a sense of incomplete evacuation” [95]. Subsequent to these criteria, the first NICE guideline on IBS was introduced in 2008 and underwent updates in 2017 [96]. As per this guideline, the diagnosis of IBS involves the presence of abdominal pain or discomfort concomitant with changes in bowel movements. Consequently, the Rome criteria were established, with the latest iteration, Rome IV criteria, unveiled in 2016 [2,97,98].

These criteria delineate IBS by characterizing abdominal pain intricately associated with disturbances in the frequency and appearance of intestinal transit. Monash University has significantly contributed to refining the diagnostic framework for IBS, defining it as a condition typified by abdominal pain or discomfort occurring at least once a week in the preceding three months, alongside fulfillment of a minimum of two of the following criteria: correlation with defecation, alterations in stool frequency, and modifications in stool consistency [19,91].

The accurate categorization of IBS into distinct types is imperative for both diagnostic precision and effective therapeutic interventions. In accordance with the 2017 guideline, IBS is stratified into three types, contingent on the dominant symptomatology: diarrhea-predominant (IBS-D), constipation-predominant (IBS-C), or alternating profiles (NICE 2008, revised 2017) [96]. However, a fourth category—unclassified IBS (IBS-U)—is also recognized, encompassing patients whose symptoms do not clearly fit into the aforementioned subtypes [91].

The incorporation of the alternating form assumes significance as it mirrors the frequent transitions of patients between different symptom profiles, thereby heightening the complexity of both diagnosis and treatment. The criteria outlined by Monash University further refine the classification of IBS, segregating it into four types based on stool pattern and bowel habit (Table 2).

In addition to the aforementioned subtypes, it is pertinent to highlight post-infectious irritable bowel syndrome (PI-IBS), a variant of IBS that manifests subsequent to an episode of infection [100]. It is imperative to precisely characterize and discern this manifestation of IBS, given that its distinct etiology may necessitate targeted anti-infective therapeutic interventions.

### 3.2. Ethiopathogenesis of IBS

The intricacy of IBS is attributed not only to its extensive and variable symptomatology but also to the incomplete understanding of its etiopathogenesis despite decades of research. The onset of the disease is influenced by a myriad of factors, encompassing genetic, nutritional, neuro-endocrine, inflammatory, psychosocial, gut–brain axis, hypothalamic–pituitary–adrenal (HPA) axis, enteric nervous system (ENS), and gut microbiota elements. Importantly, these factors are intricately interconnected and operate synergistically in the multifaceted pathogenesis of IBS [101].

In the following sections, we explore the principal categories implicated in the etiopathogenesis of IBS, beginning with genetic and microbial factors, continuing with microbially derived metabolites and their impact on motility, neuroimmune and neuroendocrine pathways, and concluding with the role of psychosocial stress and diet in symptom generation and chronicity.

#### 3.2.1. Genetic Susceptibility and Mutations Implicated in IBS

While a singular genetic determinism for IBS remains elusive, IBS patients often exhibit positive hereditary and collateral antecedents. The prevalence of the disease is notably higher in homozygous twins compared with heterozygous twin pairs [101]. Genetic investigations have identified mutations and polymorphisms in serotonin receptors, particularly the serotonin-reuptake receptor (SERT) and sucrase-isomaltase (SI), among individuals with IBS. A subset of IBS patients, approximately 2%, carries a mutation in the SCN5A gene, documented by Beyder et al. (2015), which disrupts the activity of voltage-dependent Na channels and is predominantly associated with the IBS-C subtype [102]. These findings substantiate the role of genetics in the etiopathogenesis of IBS.

#### 3.2.2. Alterations in the Gut Microbiota as a Trigger for IBS

Understanding the complexity of the intestinal microbiota has significantly advanced our knowledge of the causes and possible treatments of many diseases, including IBS. In IBS, the pivotal role of specific bacterial strains within the gut is underscored, yet it extends beyond the mere presence of individual bacterial species. The fermentation products and secondary metabolites contribute substantively to the multifactorial underpinnings of the disease [103].

While the complete involvement of the gut microbiota in the pathogenesis of IBS remains elusive, it stands as a focal point in ongoing research endeavors. The microbiota composition in IBS patients deviates from that observed in healthy individuals, as documented by Collins (2014) [104], affecting both diversity and patterns of reduction/stability [105]. Dysbiosis is a salient feature in IBS patients, with discernible alterations occurring in both the mucosa and the intestinal lumen.

Studies have reported a discernible reduction in the abundance of *Bifidobacteria*, *Bacteroidetes*, and *Faecalibacterium prausnitzii*, coupled with an elevation in *Firmicutes* and the *Firmicutes*/*Bacteroidetes* ratio within the intestinal lumen microbiota of individuals diagnosed with IBS [106,107,108]. Notably, in the microbiota of the intestinal mucosa in IBS patients, a visible decrease in *Bifidobacteria* coupled with decreased *Lactobacillus* is evident compared with healthy counterparts [108,109].

Further investigations reveal a diminished presence of *Bacteroidetes* populations and an augmentation in *Firmicutes*, *Actinobacteria*, *Ruminococcus*, *Verrucomicrobia*, and *Proteobacteria* in individuals afflicted with IBS. Specifically, IBS-D patients exhibit a reduction in *Bacteroidetes* populations, whereas an increased prevalence of *Lactobacillus* spp. is observed in IBS-C. These findings underscore the microbiota-specific alterations associated with different IBS subtypes [106,110].

At the metagenomic level, a diverse range of pathogenic microorganisms has been identified in the gastrointestinal milieu of individuals diagnosed with IBS. These include bacteria such as *Clostridium difficile*, *Escherichia coli*, *Mycobacterium avium* ssp. *paratuberculosis*, *Campylobacter concisus*, *Campylobacter jejuni*, *Chlamydia trachomatis*, *Helicobacter pylori*, *Pseudomonas aeruginosa*, *Salmonella* spp., and *Shigella* spp. as well as non-bacterial pathogens like the protozoan *Giardia lamblia* and *Noroviruses* [82]. The heterogeneous alterations in the gut microbiota contribute significantly to the dynamic and multifaceted symptoms characteristic of IBS.

Studies have unveiled a negative correlation between the concentrations of *Bifidobacteria* and *Proteobacteria*, observed in both luminal and mucosal samples, and the pain score associated with IBS [106,111]. Conversely, a positive relationship has been established between the abundance of *Ruminoccocus torques*-like organisms and the intensity of pain experienced by individuals with IBS [112].

#### 3.2.3. Microbial Metabolites and Their Impact on Intestinal Motility

**Figure 4:**
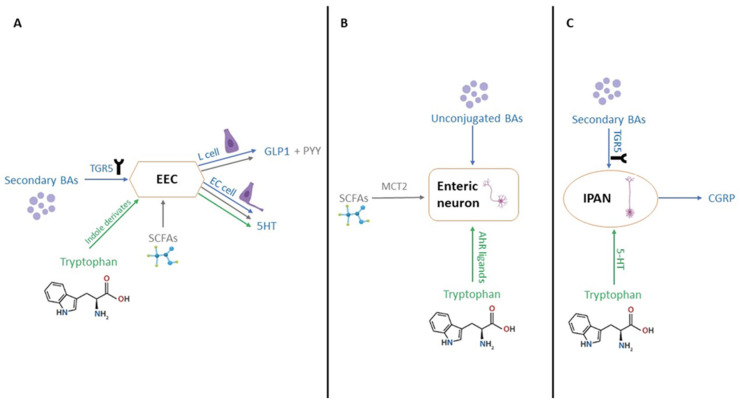
Microbial metabolites modulate gut motility and neuroendocrine signaling. (**A**) Effects of secondary bile acids, SCFAs, and tryptophan derivatives on EEC activity. (**B**) Direct activation of enteric neurons by SCFAs and AhR ligands. (**C**) TGR5-mediated signaling in IPANs via bile acids and serotonin. Created in Biorender. Varvara R. (2025) https://app.biorender.com/illustrations/689478238f728fcd8d876116 (accessed on 8 July 2025).

Recent investigations have underscored the correlation between microbiome and disease symptoms, elucidating that intestinal dysbiosis can lead to impaired intestinal motility. This impairment may occur through direct mechanisms (Figure 4A), involving the interaction of certain bacterial wall components (e.g., lipopeptides, peptidoglycan, and lipopolysaccharides) with toll-like receptors (TLRs), in particular TLR2 and TLR4, within the gastrointestinal tract [113,114]. Alternatively, dysbiosis can exert an indirect impact on motility through the modulation of secondary metabolites, including SCFAs, bile acids, and tryptophan metabolites (Figure 3 and Figure 4B) [115].

Recent studies have revealed that intestinal dysbiosis contributes to compromised intestinal motility through a myriad of mechanisms. SCFAs, primarily acetate, propionate, and butyrate play a crucial role in modulating motility by activating enteroendocrine cells (EECs) (Figure 3), inducing the production of gastrointestinal peptide-1 (GLP-1) and peptide YY (PYY) [116,117]. Additionally, they activate the 5-hydroxytryptamine 4 receptor (5-HT4) and monocarboxylate transporter 2 (MCT2) at the enteric neuron level (Figure 4B). Tryptophan, subject to the influence of gut microbiota, undergoes diverse metabolic pathways, leading to the production of aryl hydrocarbon receptor ligands (AhR ligands), tryptamine, and indole derivatives, all of which contribute to gut motility [78,79].

Lipidomic investigations have demonstrated that an altered microbiome induces both quantitative and qualitative alterations in bile acid secretion. Noteworthy, microorganisms such as *Listeria monocytogenes*, *Bacteroides vulgatus*, *Lactobacillus*, *Clostridium perfringens*, *Bifidobacterium*, and *Bacteroides fragilis* actively participate in bile acid production. The bile acid forms impacting intestinal motility encompass unconjugated bile acids and secondary bile acids, including deoxycholic acid (DCA) and lithocholic acid (LCA), both derived from the microbial impact on conjugated bile acids or primary bile acids [80]. Disruptions in gut microbiota led to disturbances in intestinal transit, as the microbiota modulates motility through unconjugated and secondary bile acids (BAs). The Taketa G-protein-coupled receptor 5 (TGR5) emerges as a pivotal mediator of intestinal motility, with DCA activating TGR5 at the level of EECs, thereby increasing GLP-1 levels and slowing transit, particularly in the ileum [81]. DCA also elevates the level of 5-hydroxytryptamine (5-HT), which governs intestinal peristaltic contractions [118,119]. Crucially, the pathways through which bile acids and tryptophan metabolites influence intestinal motility intersect. TGR5 activation extends to intrinsic afferent primary neurons (IPANs) (Figure 4C), capable of producing calcitonin gene-related peptide (CGRP) and thereby modulating intestinal motility. Unconjugated bile acids directly stimulate enteric neurons, exerting an additional impact on motility [120]. In individuals with IBS-D, the reduction in the *Ruminococccaceae* family leads to an increase in the concentration of primary bile acids and a concurrent decrease in the concentration of secondary bile acids (Figure 4) [121].

The production of gases such as hydrogen, methane, and carbon dioxide, generated through the microbial fermentation of indigestible carbohydrates, affects intestinal motility by altering transit time, promoting luminal distension, and modulating the perception of visceral sensations [122].

Beyond these, other microbiota-derived products such as biogenic amines and microbial neurotransmitters also contribute to dysmotility in IBS. Histamine, a biogenic amine synthesized via histidine decarboxylation by species like *Escherichia coli* and *Morganella morganii*, can alter gastrointestinal motility, mucosal permeability, and ion transport. Elevated colonic histamine levels and increased expression of histamine receptors H1R and H2R have been reported in IBS patients, while *Lactobacillus reuteri* has been shown to exert anti-inflammatory effects via histamine-mediated H2R activation [123].

Additionally, gut bacteria influence the local and systemic availability of neurotransmitters such as γ-aminobutyric acid (GABA), serotonin, dopamine, and norepinephrine, impacting both the enteric and central nervous systems. These interactions underscore the neuroactive potential of microbial metabolites in shaping motility, pain perception, and the overall clinical picture of IBS [123].

#### 3.2.4. Microbiota–Gut–Brain Axis and Visceral Pain Modulation

Recent investigations have brought to light the intricate involvement of the gut microbiota in the pathogenesis of irritable bowel syndrome (IBS), prompting a paradigm shift from the traditional gut–brain axis to the emerging microbiota-gut–brain (MGB) axis [114]. This innovative conceptualization of the MGB axis posits that metabolic and biochemical processes within the gut microbiota play a pivotal role in maintaining homeostasis through bidirectional communication involving the gut, brain, and immune system, allowing microbial metabolites to influence host behavior, mood, motility, and visceral pain perception [6,114].

In the context of IBS, disruptions in the communication between the vagus nerve and the intestinal microbiota result in alterations along the MGB pathway. The influence of gut microbes on vagus nerve function is mediated through diet-responsive metabolites, special SCFAs, and a variety of neuroactive compounds such as serotonin, dopamine, acetylcholine, glutamate, γ-aminobutyric acid (GABA), and noradrenaline. These molecules impact the motor and secretory functions of the intestine, dependent on synapses between vagus nerve endings and the enteric nervous system, affecting motility, immunity, and sensory processing [84,114].

SCFAs have been shown to play a critical role in the modulation of visceral pain. This effect is mediated by the activation of G-protein coupled receptors such as GPR43 and GPR41, located on enteric neurons and immune cells [124]. In addition, SCFAs influence the synthesis and release of neurotransmitters involved in pain regulation, including GABA and serotonin, thereby contributing to the sensory symptoms of IBS [125].

Serotonin (5-HT) mediates visceral hypersensitivity, being synthesized in proportion to 90% by gut bacteria. Elevated levels of 5-HT may sensitize afferent neurons and amplify nociceptive signaling, contributing to the pain symptoms of IBS [126].

#### 3.2.5. Inflammation and Intestinal Barrier Dysfunction in IBS

The exploration of the gut microbiota’s involvement in IBS pathogenesis has illuminated the interplay between the immune system and disease development. Microbiota alterations affect the integrity of the intestinal mucosa, inducing a state of chronic inflammation. This emphasizes the necessity of investigating the nexus between immunity and IBS, even in transient changes [104].

While inflammation is a common feature across all IBS types, it is particularly pronounced in the post-inflammatory subtype, potentially elucidating symptom variability and a higher prevalence among women [5]. The precise mechanism by which inflammation triggers IBS symptoms remains incompletely understood, with proposed hypotheses including changes in intestinal permeability leading to “leaky gut syndrome” and an elevated expression of toll-like receptors [127]. Once established, leaky gut syndrome can initiate and sustain inflammation, marked by increased synthesis of pro-inflammatory cytokines such as interleukin-6 (IL-6) and interleukin-8 (IL-8). These cytokines contribute significantly to the overall pathogenesis and symptomatology of IBS [128,129].

In individuals with IBS, there is an observed elevation in the number of mast cells proximate to intestinal sensory neurons, exhibiting a direct correlation with the frequency and intensity of painful episodes. The infiltration of mast cells into the colonic mucosa has a direct impact on the enteric nervous system, contributing significantly to the perception of pain [130]. Notably, this mast cell infiltration is more conspicuous in IBS-D and is akin to that observed in ulcerative colitis during remission. Dysbiosis emerges as a pivotal factor in the genesis of leaky gut syndrome, a condition associated with mast cell activation and reduced pain thresholds [131]. Additionally, the involvement of *Campylobacter pylori* has been implicated in the genesis of leaky gut syndrome [132]. These insights provide a framework for understanding the heightened perception of certain sensations as painful in IBS patients, even in the absence of apparent physiological triggers.

#### 3.2.6. The Role of Stress and Psychosocial Triggers in IBS

Psychological stress is increasingly recognized as a key factor in the onset and exacerbation of IBS. Both acute and chronic stress have been shown to influence gastrointestinal function through central and peripheral pathways, including activation of the hypothalamic–pituitary–adrenal (HPA) axis, modulation of gut motility, altered visceral sensitivity, and disruption of intestinal permeability [133].

The heightened secretion of interleukin-6 (IL-6) in the intestinal mucosa by *Aspergillus fumigatus*, *Candida albicans*, and *Saccharomyces cerevisiae* has been implicated in activating the HPA axis. This activation results in an upsurge in cortisole synthesis, linking it to the onset of depression, especially in women [134]. Cortisol affects the gut’s physiology by increasing intestinal permeability, altering mucosal immune responses, and promoting pro-inflammatory signaling, which may contribute to symptom generation and persistence in IBS. Patients with IBS frequently report higher levels of anxiety, depression, and somatization [135,136] and they also display alterations in the *Firmicutes*/*Bacteroidetes* ratio within the lumen and an increased abundance of mucosal *Escherichia coli*. These observations underscore the pivotal role of the gut microbiota in modulating HPA-axis activity, contributing to the development of psychological disorders in individuals with IBS. Psychological interventions, including cognitive behavioral therapy (CBT), hypnotherapy, and mindfulness-based stress reduction, have shown efficacy in symptom improvement, further supporting the pathogenic relevance of psychosocial stressors [137].

#### 3.2.7. The Role of Diet in IBS Pathogenesis

Examining IBS through the lens of altered microbiota underscores the central role of dietary factors in both the etiology and management of the condition. The exacerbation or onset of symptoms following the consumption of specific foods or food groups, coupled with symptom amelioration upon their exclusion, serves as a critical indicator of the substantial impact of diet on the pathogenesis and progression of the disease. Among the implicated food components are short-chain carbohydrates (fructose, lactose, galactose), sugar alcohols, and fructans [4,99]. These indigestible carbohydrates exert an osmotic effect in the colon and undergo fermentation by gut microflora, leading to symptoms such as abdominal pain, bloating, distension, and flatulence [138]. The influence of dietary elements and/or stressors on gastrointestinal function contributes to aberrant motility, thereby substantiating the hypothesis implicating dietary patterns in the pathogenesis of IBS.

**Figure 5:**
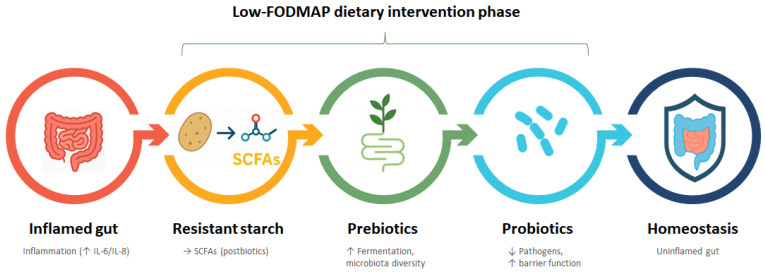
Diet–Microbiota Axis: Nutritional progression toward gut homeostasis. Stepwise illustration of the Low-FODMAP dietary intervention phase aimed at restoring gut homeostasis. The process begins with an inflamed gut, characterized by elevated inflammatory markers (↑ IL-6/IL-8). Introduction of resistant starch promotes the production of beneficial postbiotic metabolites, particularly short-chain fatty acids (SCFAs), which support anti-inflammatory effects. This is followed by the administration of prebiotics, which enhance fermentation and microbiota diversity. Subsequently, probiotics are introduced to reduce pathogenic bacteria and improve gut barrier function. The intervention culminates in gut homeostasis, marked by reduced inflammation and improved intestinal integrity. The arrows represent the sequential transition between each intervention step, indicating a progressive and synergistic approach in the dietary strategy—from inflammation to restored gut health—through the combined use of resistant starch, prebiotics, and probiotics. Created in PresentationGO. Heghes S.C. (2025) https://PresentationGO.com (accessed on 8 July 2025).

Personalized nutritional therapy, based on the exclusion of specific food groups such as fructans, polyols, and fructo-oligosaccharides, constitutes a pivotal non-pharmacological modality for treating IBS. These dietary measures are integral to the fermentable oligosaccharides, disaccharide, monosaccharides, and polyols (FODMAP) diet, recognized as one of the most efficacious therapeutic and dietary approaches for managing IBS [139]. The effectiveness of this nutritional intervention has been substantiated both subjectively, through symptom improvement, and objectively, through the reduction in pro-inflammatory cytokines such as IL-6 and IL-8 (but not TNF-alpha) following a minimum three-week adherence to the FODMAP diet (Figure 5) [4,140]. The implementation of the low-FODMAP diet typically follows a structured three-phase protocol: an initial strict restriction phase lasting 2 to 6 weeks, followed by a systematic reintroduction phase to identify individual triggers, and a personalized maintenance phase. Commonly restricted foods include garlic, onions, wheat, legumes, apples, pears, and dairy products containing lactose. While clinical improvements are often observed, prolonged adherence without professional supervision may lead to reduced microbial diversity, notably a decrease in Bifidobacteria and butyrate-producing taxa [99]. Therefore, the reintroduction and diversification of fermentable fibers—such as RS and other prebiotics—are essential for restoring gut microbial balance and long-term symptom control [141].

Dietary interventions in the management of IBS aim to eradicate triggering factors, such as fructans, polyols, and fructo-oligosaccharides, while concurrently introducing supportive elements like RS, prebiotics, and probiotics [4]. The following sections will explore how resistant starch, through its impact on microbiota-derived metabolites, may contribute to novel therapeutic avenues in IBS.

## 4. RS in IBS—Mechanisms and Clinical Application

### 4.1. Practical Use of Resistant Starch in IBS Management

A growing number of clinical and meta-analytic evidence supports the efficacy of RS, particularly types RS2 and RS3, in the dietary management of IBS. As summarized in Table 3, randomized controlled trials (RCTs) and systematic reviews demonstrate improvements in fecal bulk, microbiota composition, inflammatory markers, and gastrointestinal symptoms across various IBS subtypes.

While the recommended daily intake of resistant starch ranges from 10 to 45 g depending on the IBS subtype and individual tolerance [143,145], this amount is part of the broader dietary fiber (DF) recommendation. According to EFSA, an adequate intake of DF for adults is set at 25 g/day [148]. This total includes various fiber fractions—such as cellulose, hemicellulose, pectins, β-glucans, and lignin—each contributing differently to gut function. Compared with other fiber types, RS exerts more specific effects on colonic fermentation and postbiotic production, particularly increasing butyrate levels, which are especially relevant in IBS management [149]. Although most clinical trials support the tolerability and microbial modulation of RS2 and RS3, short-term interventions may not be sufficient to produce meaningful symptom improvement in IBS patients. For instance, So et al. (2022) conducted the first double-blind RCT in Rome IV-confirmed IBS subjects and found that a 7-day intervention with 20 g/day of RS2 did not significantly improve gastrointestinal symptoms, stool characteristics, or SCFA levels [147]. This highlights the importance of both treatment duration and individualized responses in RS-based interventions.

The therapeutic potential of resistant starch (RS) has been explored across the various clinical subtypes of IBS, with promising results in terms of symptom modulation, microbiota shifts, and inflammatory markers. The type and dosage of RS, as well as the predominant pathophysiological mechanism in each IBS subtype, appear to influence the observed outcomes.

In IBS-D (diarrhea-predominant), RS2 and RS3 have demonstrated efficacy in improving stool consistency and reducing inflammatory activity. Meta-analytic data from Shen et al. reported increased fecal weight following RS supplementation, while Vahdat et al. showed significant reductions in pro-inflammatory markers such as IL-6 and TNF-α [143,145]. Correlative analyses by Bush et al. further supported the microbiota–symptom linkage in this subgroup.

For IBS-C (constipation-predominant), RS2 and RS3 have shown consistent bulking and motility-enhancing effects. In addition to RS2, RS3 has also demonstrated clinical relevance in IBS-C. Jenkins et al. [88] compared RS2 and RS3, observing a 31% increase in SCFA levels and enhanced fecal volume. More recently, Luk-In et al. [86] administered RS3 at 9 g/day in Thai adults with functional constipation, reporting significant improvements in stool consistency and increases in beneficial taxa such as Bifidobacterium and Akkermansia. These findings support the applicability of RS3 in improving bowel function and microbiota profiles in IBS-C patients.

In IBS-M (mixed), the modulation of microbial diversity and resilience has been highlighted. Supplementation with RS blends (RSB) has been associated with increased *Faecalibacterium* abundance [146], individualized microbial response patterns [87], and the potential for personalized treatment, as illustrated by Dobranowski et al. [150].

Patients classified as IBS-U (unclassified) may also benefit from RS3, particularly in cases where low-grade inflammation plays a role. Evidence from IBD cohorts [142] and meta-analyses reporting reductions in inflammatory mediators [143] suggest overlapping mechanisms relevant to IBS pathophysiology.

The implementation of resistant starch (RS) therapy in individuals with irritable bowel syndrome should follow a structured evidence-based protocol to optimize tolerability and efficacy. During the initial adaptation phase (weeks 0–4), it is recommended to initiate treatment with a starting dose of 5–10 g/day, allowing for progressive adaptation of the gut microbiota and minimizing the risk of fermentation-related adverse effects [146,147]. Gastrointestinal tolerance should be closely monitored, with dose adjustments made as necessary. In the subsequent dose optimization phase (weeks 4–8 to 12), RS intake can be gradually increased, typically in increments of 5 g every 2 weeks, until reaching a target dose of 15–30 g/day, depending on individual response and clinical goals [151]. Throughout this period, practitioners should assess stool characteristics using validated tools such as the Bristol Stool Scale, alongside relevant inflammatory markers (e.g., IL-6, fecal calprotectin) and patient-reported outcomes regarding symptom control and quality of life [86,152,153]. The maintenance phase (beyond week 8–12) involves continuing RS administration at the most effective dose established during the previous stage. Long-term response should be evaluated periodically every 2–3 months, with dose adjustments or re-evaluation of the microbiota profile considered in cases of clinical plateau or symptom recurrence [154].

While current clinical trials have primarily targeted adult populations with mild to moderate IBS, special consideration should be given to vulnerable groups who may exhibit distinct microbiota profiles or altered gastrointestinal physiology. However, special consideration is warranted for vulnerable populations—such as the elderly and children—due to their distinct microbiota composition, immune maturity, and gastrointestinal physiology, which may influence both the tolerability and metabolic outcomes of RS intervention. In elderly patients, age-related dysbiosis and slower colonic transit may influence RS fermentation and metabolite production, potentially enhancing therapeutic efficacy but also increasing sensitivity to initial gastrointestinal symptoms [155,156]. In pediatric populations, although data remain limited, the modulation of developing microbiota through RS intake may offer long-term benefits. However, evidence from clinical studies remains inconclusive. For instance, a 4-week RS2 intervention in Malawian undernourished children aged 3–5 years led to increased propionate levels and microbial shifts but also elevated fecal calprotectin without overt symptoms, suggesting possible subclinical intestinal activation [157]. These findings underscore the need for cautious dosing, clinical monitoring and further studies to assess both efficacy and safety in this vulnerable population [158]. Individuals with comorbid metabolic disorders, such as obesity, insulin resistance, or dyslipidemia, may benefit from the dual effects of RS on glycemic control and gut microbiota balance. In this context, acute dietary interventions in healthy adults have shown that RS4 can reduce postprandial glycemia and insulinemia compared with rapidly digestible starches, supporting its potential use in IBS patients with insulin resistance or metabolic dysfunctions [151]. Moreover, patients with IBS and coexisting anxiety or depression may be responsive to RS-derived postbiotics acting on the gut–brain axis, such as GABA and tryptophan metabolites [9,115]. These considerations emphasize the need for stratified approaches in RS therapy and further research to validate safety and efficacy in these populations.

Resistant starch is generally recognized as a safe and well-tolerated dietary component. Nevertheless, during the initial phase of fermentation, certain transient gastrointestinal symptoms may occur. The most frequently reported effects include flatulence and bloating, observed in more than 10% of users, as well as mild abdominal discomfort or cramping, with a moderate incidence ranging between 1% and 10% [147,158]. To improve tolerability, several strategies may be employed, such as initiating with lower doses and increasing gradually, fractioning the daily intake, or combining RS with probiotics to enhance microbial resilience. In sensitive individuals, the use of digestive enzymes or antispasmodics may further alleviate discomfort. RS supplementation is contraindicated in patients with intestinal obstruction, severe malabsorption syndromes, or known hypersensitivity to the starch source (e.g., maize, potato, or tapioca) [150]. A thorough assessment of medical history is thus essential prior to initiating therapy.

### 4.2. Personalized Approaches in RS Therapy

The therapeutic use of resistant starch (RS) in irritable bowel syndrome (IBS) requires a nuanced approach tailored to the individual’s symptom profile, microbial composition, and metabolic background. While standard protocols provide a general framework, personalization has the potential to optimize outcomes and minimize adverse effects.

Variations in the gut microbiota significantly influence the response to RS. Individuals with a *Bacteroides*-dominant enterotype appear to benefit more from RS2 sources such as raw potato starch, whereas those enriched in *Prevotella* spp. may respond preferentially to RS3 derived from retrograded starchy foods [150,159]. The presence of key degraders, such as *Ruminococcus bromii*, has been consistently associated with improved fermentation capacity and the increased production of butyrate and other short-chain fatty acids (SCFAs) [69]. These microbial signatures may inform the choice of RS formulation, as well as the expected trajectory of symptom relief.

Genetic variability also plays a role in shaping individual responses to RS. For example, polymorphisms affecting salivary amylase gene copy number (AMY1) may alter starch digestion kinetics, thereby influencing the amount of RS that reaches the colon [160]. Similarly, host variants in G-protein coupled receptors (e.g., GPR41, GPR43) that sense SCFAs can modulate downstream metabolic and immunological effects, further contributing to interindividual differences in RS efficacy and tolerability [63].

The relationship between resistant starch (RS) and the gut microbiome is inherently bidirectional, forming a dynamic feedback loop that underpins its therapeutic potential. On one hand, RS—especially RS3 and RS2—modulates the composition and function of the microbiota by enriching beneficial taxa such as *Faecalibacterium prausnitzii*, *Ruminococcus bromii*, *Bifidobacterium* spp., and *Akkermansia muciniphila*, which are key producers of butyrate and other health-promoting metabolites [64,69,86,146]. On the other hand, the structure and functionality of RS is transformed by the enzymatic machinery of the microbiota, which hydrolyzes RS through specialized amylases and glycosidases, producing a spectrum of bioactive fermentation products including SCFAs (e.g., butyrate and propionate), indole derivatives, GABA, and secondary bile acids [64,68,73,78,81]. These postbiotics, in turn, reshape microbial ecology by altering pH, cross-feeding dynamics, and signaling through receptors such as GPR43, GPR41, and TGR5 [63,65,68,123]. For instance, butyrate production not only supports colonic epithelial integrity but also feeds back to suppress pro-inflammatory taxa and reinforce colonization of anti-inflammatory commensals [64,69,71]. Therefore, RS acts as both a driver and a responder within the gut ecosystem, enabling a personalized, microbiota-mediated route for the generation of functionally relevant bioactives in IBS and other dysbiosis-related conditions [36,69,150].

In clinical practice, personalization can be achieved by integrating basic patient profiling with accessible markers. Identification of IBS subtype, titration of RS dose based on stool characteristics, and, where feasible, assessment of microbial composition can guide therapeutic decisions. For patients experiencing suboptimal outcomes, a switch in RS type or co-administration with probiotics may enhance tolerability and clinical benefit. Incorporating microbial and host-related factors into RS supplementation strategies may support the development of precision nutrition models for IBS management, allowing interventions to be tailored to the underlying biological context of each patient.

### 4.3. Comparative Perspective: Resistant Starch, FODMAP Diet, Prebiotics, and Related Strategies

As previously discussed in Section 3.2.7, the FODMAP diet plays a central role in the dietary management of IBS through modulation of intestinal fermentation and osmotic load [4]. However, in clinical practice, it must be considered alongside other interventions such as resistant starch, prebiotics, and probiotics, each with distinct benefits and limitations. A comparative overview is provided below. Multiple nutritional strategies have been proposed for the management of IBS, each with distinct mechanisms of action, levels of evidence, and practical limitations. Among these, RS, the FODMAP diet, prebiotics, probiotics, and soluble fibers represent complementary or alternative tools in clinical practice.

RS, particularly type III, exerts its effects via slow colonic fermentation, leading to the gradual production of SCFAs and favorable modulation of the gut microbiota. Its tolerability in IBS is generally good, with fewer reports of gas-related symptoms compared with other fermentable substrates [147]. However, clinical evidence remains moderate, and sustained benefits depend on continued intake.

The FODMAP diet continues to play a central role in IBS dietary therapy, due to its high short-term efficacy, reported to improve symptoms in approximately 70% of patients [4,161]. Its primary mechanism involves reducing the fermentative load and osmotic activity of fermentable sugars [138]. Nevertheless, its restrictive nature may alter microbiota diversity if maintained long-term, and its implementation often requires professional guidance [162].

Probiotics act through direct modulation of microbial communities, with efficacy highly dependent on strain selection. While well-tolerated in many cases, they may require prolonged administration and have variable results across IBS subtypes [108].

Prebiotics such as fructooligosaccharides (FOS) and galactooligosaccharides (GOS) selectively stimulate beneficial bacteria but may cause adverse gastrointestinal effects, particularly in IBS-D or in those with visceral hypersensitivity [12]. Soluble fibers, by contrast, improve stool consistency and transit time, with moderate efficacy and good tolerability, though their benefits are limited to the period of active use [139].

### 4.4. Future Perspectives on Resistant Starch in IBS Management

Despite the promising physiological and microbial effects associated with resistant starch (RS), its role in the dietary management of irritable bowel syndrome (IBS) remains incompletely elucidated. Variability in individual tolerance, diversity in RS types, and interactions with the gut microbiota warrant the development of targeted strategies that integrate RS into IBS therapy. Future research efforts should address several key areas to ensure evidence-based and personalized implementation [150].

#### 4.4.1. Long-Term Clinical Evaluation in IBS Populations

To date, most clinical studies investigating RS and gastrointestinal health have been short-term and focused primarily on healthy subjects or general metabolic outcomes [29]. There is a critical need for long-duration randomized controlled trials (RCTs) in IBS populations, with follow-up periods of at least 12 months to assess sustained symptom relief, microbial shifts, and host adaptation. Endpoints should include validated symptom scores, stool consistency (e.g., Bristol Scale), quantitative SCFA profiles, and health-related quality of life (QoL) metrics. Stratification by IBS subtype (IBS-C, IBS-D, and IBS-M) and microbiota enterotypes may allow for more refined outcome interpretations. Adaptive trial designs with dose titration and parallel microbiome–metabolome assessments are encouraged [86].

#### 4.4.2. Standardization and Optimization of RS Preparations

The heterogeneity of RS sources and types poses a significant challenge to reproducibility and clinical translation. Future studies should focus on the systematic analytical characterization of RS used in trials, including structural conformation, the degree of polymerization, enzymatic resistance, fermentation rate, and SCFA production profiles. A priority remains in the development of standardized, well-tolerated RS formulations, including enteric-coated powders, low-FODMAP-compliant functional foods enriched with RS, and gastro-resistant capsules [162]. Moreover, RS5, a structural variant formed via complexation with lipids, deserves further exploration due to its altered fermentability and postbiotic profile [163]. Regulatory frameworks may need to be updated to account for such formulations as medical foods or functional adjuvants in IBS therapy.

#### 4.4.3. Integration with Multimodal and Synbiotic Therapies

Given the multifactorial pathophysiology of IBS, RS may be most effective when included in combined therapeutic strategies. A promising direction involves synbiotic combinations pairing RS with butyrate-producing or spore-forming probiotics (e.g., *Clostridium butyricum* and *Faecalibacterium prausnitzii*) to enhance colonic fermentation while mitigating gas-related side effects [150]. Additionally, co-administration with polyphenols could potentiate microbial diversity and beneficial fermentation, while protecting against oxidative stress and inflammation. RS can also be integrated into low-FODMAP diets, where its selective fermentability supports microbial restoration without symptom flares [164]. Emerging approaches within gut–brain axis therapies, including psychobiotics (e.g., *Lactobacillus rhamnosus*, *Lactobacillus helveticus*, *Lactobacillus plantarum*, *Bifidobacterium longum*, *Bifidobacterium infantis*, and *Lactobacillus casei*), gut-directed hypnotherapy, and mindfulness-based stress reduction, may benefit from RS inclusion, given its role in generating microbial neurotransmitters (e.g., GABA and serotonin), volatile organic compounds, and anti-inflammatory peptides that influence neuroimmune signaling [123,165].

Resistant starch, particularly type III (RS3), can be conceptualized not only as a fermentable dietary fiber but also as a smart, site-specific delivery system for modulating host–microbiota interactions [33,35,36]. Due to its structural resistance to upper gastrointestinal digestion, RS3 reaches the colon intact, where it undergoes targeted microbial fermentation [29,31,36]. This delayed-release behavior enables RS3 to function as a microbiota-targeted substrate, driving the production of bioactive metabolites such as short-chain fatty acids (SCFAs), indole derivatives, bile acid metabolites, and neuroactive compounds like GABA and serotonin precursors [64,68,78,81,123]. These postbiotics act locally and systemically to modulate inflammation, enhance epithelial barrier function, reduce visceral sensitivity, and influence gut–brain axis signaling [64,69,72,78,123,129]. Therefore, RS3 mimics controlled-release delivery systems, offering a functional nutritional scaffold for therapeutic and preventive interventions in IBS and other microbiota-associated conditions [29,36,150].

In addition to its intrinsic fermentability, resistant starch (RS)—particularly RS3 and RS5—can be further optimized through innovative delivery technologies that enhance its colonic targeting, microbial interaction, and clinical efficacy. Microencapsulation of RS using gastro-resistant coatings or biopolymer matrices has been explored to protect its structure during gastric transit and ensure site-specific release in the ileocolonic region, where microbial density and fermentation capacity are highest [36,55]. Moreover, co-administration with butyrate-producing probiotics such as *Faecalibacterium prausnitzii*, *Clostridium butyricum*, or *Roseburia* spp. offers a synergistic approach to maximize SCFA output and reinforce mucosal immune modulation [69,150]. These synbiotic strategies, especially when combined with minimally fermented fibers or polyphenols, can also buffer against transient fermentation-related discomfort by promoting microbial resilience and cross-feeding [146,150]. Furthermore, nanostructured RS delivery systems, where RS functions as a prebiotic scaffold for encapsulated probiotics and bioactives (e.g., polyphenols or bile acid-modulating agents), are gaining interest as next-generation approaches to modulate gut microbiota composition and host response in a personalized manner [20,163]. These layered and co-formulated delivery strategies not only improve functional outcomes but also align with emerging trends in precision gut-targeted therapies and microbiome-based nutrition interventions.

#### 4.4.4. Toward Personalized Nutrition: Stratification and Predictive Models

Interindividual differences in the physiological and microbial response to resistant starch (RS) highlight the necessity of adopting precision nutrition strategies. To optimize clinical outcomes, a comprehensive integration of multi-omics data (such as gut microbiota composition, SCFAs profiles, inflammatory cytokine levels, and intestinal permeability biomarkers) [166,167,168], and host genetic variants (such as AMY1 copy number and SCFA receptor polymorphisms) [169] may enable the stratification of individuals according to their expected responsiveness to RS. Such data-driven approaches could inform tailored dietary interventions, accounting for both microbial functionality and host metabolic signatures.

To advance toward precision nutrition in IBS, it is essential to stratify patients according to microbiome composition, functional capacity, and host genetic markers that modulate the fermentation of and clinical response to resistant starch (RS). Specific microbial taxa such as *Ruminococcus bromii*, *Bifidobacterium adolescentis*, and *Faecalibacterium prausnitzii* are key RS degraders and butyrate producers, and their baseline abundance correlates with enhanced SCFA generation and symptomatic improvement [64,69,150]. Enterotype classification—particularly the dominance of *Bacteroides* versus *Prevotella*—has also been associated with distinct fermentation patterns and responsiveness to RS2 versus RS3 [150,159]. On the host side, genetic variations such as AMY1 gene copy number, which influences salivary starch digestion and colonic substrate availability and polymorphisms in SCFA receptors (e.g., GPR41 and GPR43), may affect the metabolic and immune response to RS-derived metabolites [63,160]. Moreover, IBS-C and IBS-M subtypes appear to respond more favorably to RS3 supplementation, likely due to its bulking, microbiota-stabilizing, and butyrogenic effects [86,88,146]. Integrating these host and microbial features into a predictive model—a proposed “RS Response Score”—could facilitate personalized interventions that match RS type, dosage, and co-treatments (e.g., synbiotics or polyphenols) to the patient’s biological profile. Such stratification frameworks could ultimately increase therapeutic efficacy while minimizing side effects and interindividual variability [63,150,159].

Machine learning algorithms could be leveraged to generate clinical decision-support tools capable of recommending RS type, dose, and co-ingredients tailored to individual needs. These tools may be particularly relevant in complex IBS phenotypes, such as post-infectious IBS, IBS overlapping with small intestinal bacterial overgrowth SIBO, anxiety-related symptomatology, or psychosomatic-dominant subtypes [170,171].

Finally, the current technological classification of RS (RS1–RS5) does not reflect functional outcomes. A future goal should be the development of a functional classification scheme based on fermentability, postbiotic profiles, and clinical response markers. Such a classification would guide product design and therapeutic targeting more effectively.

## 5. Limitations and Gaps

Despite the growing body of evidence supporting the beneficial effects of RS on gut health and symptom modulation in IBS, several limitations and research gaps remain that warrant consideration. A significant proportion of existing data is derived from preclinical or in vitro studies, while human trials remain limited in number, scale, and methodological consistency. Clinical investigations to date have employed heterogeneous designs, with variations in RS types (e.g., RS2, RS3, and RS4), dosages (ranging from 5 to 40 g/day), and intervention durations (from two weeks to several months). These disparities complicate the aggregation and comparison of results, limiting the formulation of standardized therapeutic protocols [138,172].

Moreover, the lack of consensus on validated gut health biomarkers and outcome measures impedes the translation of mechanistic findings into practical clinical recommendations. While fecal SCFA levels, inflammatory markers (e.g., IL-6, calprotectin), and microbial diversity indices have been proposed, their utility across diverse IBS phenotypes remains uncertain [173].

An additional limitation lies in the incomplete understanding of the molecular mechanisms by which RS and its fermentation products (such as butyrate, indoles, and secondary bile acids) exert systemic effects. Further elucidation of these pathways is crucial, particularly in relation to host immune modulation and gut–brain axis signaling.

Importantly, the variability in individual response represents a critical challenge. Genetic factors, including AMY1 gene copy number and polymorphisms in SCFA receptors (GPR41, GPR43), as well as baseline microbial composition and functional enterotypes, may significantly influence the metabolic processing and clinical efficacy of RS. Lifestyle factors such as stress levels, dietary patterns, and physical activity further modulate this response, highlighting the need for a personalized therapeutic framework [165].

However, implementing personalized RS therapy presents practical constraints. The costs for multi-omics profiling remain high, with microbiome sequencing and host genotyping often exceeding the range feasible for routine use. Future research must aim to simplify and reduce the cost of predictive testing to enable broader implementation.

Finally, compliance and acceptability remain pivotal concerns. Gastrointestinal side effects such as flatulence and bloating are common during the initial stages of RS supplementation, affecting up to 70% of users. The organoleptic properties of RS-enriched foods, such as taste, texture, and appearance, may reduce patient adherence, particularly in long-term regimens. Addressing these issues through improved formulation technologies and targeted patient education strategies is essential for sustained therapeutic success [146].

Collectively, addressing these limitations through rigorous, large-scale, and standardized clinical trials—coupled with a systems biology approach—will enhance our understanding of RS functionality and its integration into evidence-based IBS management.

## 6. Conclusions

Resistant starch (RS) represents a next-generation dietary component that acts as a microbiota-targeted delivery system for bioactive metabolite generation. Once fermented in the colon, RS yields functionally diverse postbiotics—such as butyrate, indole derivatives, GABA, and secondary bile acids—which collectively influence gut–immune signaling, epithelial integrity, and visceral sensitivity. These effects are not uniform but are instead shaped by interindividual differences in gut microbiota composition, metabolic capacity, and host genetic variants such as AMY1 copy number and SCFA receptor polymorphisms.

Clinical and mechanistic evidence suggests that RS—especially RS3—may benefit specific IBS subtypes (e.g., IBS-C and IBS-M), but personalization remains critical. Integrating microbiome profiling and stratified delivery strategies (e.g., RS + synbiotics or polyphenols) offers a pathway toward precision nutritional therapy. In conclusion, RS bridges dietary intervention with microbiome science, supporting the design of personalized, microbiome-informed interventions that enhance therapeutic outcomes in IBS and potentially other microbiota-related conditions. However, current evidence is limited by the short duration of most clinical interventions, small sample sizes, and considerable heterogeneity across study designs and IBS subtypes. These factors hinder the generalizability and translational value of findings. Moreover, vulnerable populations such as children and elderly individuals remain underrepresented, despite their distinct physiological and microbial profiles. Future research should address these gaps by implementing longer-term, stratified trials with standardized endpoints, incorporating microbiome-based biomarkers, and exploring synergistic combinations of RS with complementary modulators such as polyphenols or probiotics.

## Figures and Tables

**Table 1:** Types of Resistant Starch (RS) [29,31,32,33].

Type	Description	Sources/Examples
RS1	Physically inaccessible starches due to encapsulation within cell walls or fibrous matrices	Whole or partially milled grains and seeds
RS2	Native granular starches with a compact crystalline structure resistant to enzymatic digestion	Raw potatoes, green bananas, high-amylose maize
RS3	Retrograded starches formed after gelatinization and cooling, leading to recrystallization	Cooked and cooled pasta, rice, potatoes
RS4	Chemically modified starches via cross-linking or substitution to enhance resistance	Commercially modified starches in processed foods
RS5	Amylose–lipid complexes formed during cooking or processing, increasing structural resistance	Cooked foods containing amylose and lipids

**Table 2:** Diagnostic criteria for the four subtypes of IBS, as defined by Rome IV and adopted by Monash University [19,96,99].

Subtype	Diagnostic Criteria
IBS-C (Constipation-predominant)	>25% of bowel movements with Bristol stool types 1 or 2 and <25% with types 6 or 7
IBS-D (Diarrhea-predominant)	>25% of bowel movements with Bristol stool types 6 or 7 and <25% with types 1 or 2
IBS-M (Mixed type)	>25% of bowel movements with Bristol stool types 1 or 2 and >25% with types 6 or 7
IBS-U (Unclassified)	Meets diagnostic criteria for IBS, but stool patterns do not clearly fit into the categories above

Note: The NICE guideline (2008, revised 2017) refers primarily to IBS-C, IBS-D, and IBS-M, but the unclassified form (IBS-U) is acknowledged in international frameworks such as Rome IV.

**Table 3:** Clinical studies on RS use in IBS.

Year	Design	Studies/Participants	Intervention	Main Results	References
2020	SR + MA	21 preclinical + 7 clinical	RS vs. control in IBD	SMD −1.83 mucosal damage; positive effects in all clinical studies	[142]
2020	SR + MA	13 studies, 14 effect sizes	RS vs. control	IL-6: −1.11 pg/mL; TNF-α: −2.19 pg/mL; CRP: NS	[143]
2021	SR + MA	19 RCTs	RS vs. digestible starch	Glucose: −0.09 mmol/L; HOMA-IR: −0.33	[144]
2017	SR + MA	13 RCTs	RS vs. control in healthy	Fecal weight: +35.51 g/day; Butyrate: SMD 0.61; Fecal pH: −0.19	[145]
2024	RCT double-blind	87 Thai adults with chronic constipation	RS3 9 g/day vs. placebo	BSS score ~4 at 6 weeks; ↑*Bifidobacterium*, *Prevotella*, *Akkermansia*	[86]
2024	Secondary analysis RCT	70 healthy Canadian adults	RPS 3.5–7 g/day vs. placebo	21 significant bacteria–symptom correlations; personalized response patterns	[87]
2022	RCT innovative	Healthy adults	RS blends 0–30 g/day + smart caps	GI effects with 5 g RS/day; ↑*Faecalibacterium*, *Akkermansia*	[146]
2022	RCT pilot, double-blind, cross-over	40 IBS patients (Rome IV)	RS2 20 g/day, RS2 + minimally fermented fiber (PGX; 5 g/day) vs. placebo	RS2: ↑flatulence, no symptom change; RS2 + PGX: good tolerability, no SCFA effect	[147]
1998	RCT crossover	24 subjects	RS2, RS3 vs. control	Butyrate: SCFA + 31%; Fecal volume +22 g/day	[88]

Abbreviations: SR—Systematic Review; MA—Meta-Analysis; RCT—Randomized Controlled Trial; ↑—increase; PGX—PolyGlycopleX^®^: a proprietary formulation consisting of three highly purified, water-soluble polysaccharides (minimally fermented fiber): konjac powder, sodium alginate, and xanthan gum.

## Data Availability

No new data were created or analyzed in this study. Data sharing is not applicable to this article.

## Abbreviations

The following abbreviations are used in this manuscript:

RS	Resistant starch
IBS	Irritable bowel syndrome
SCFA	Short-chain fatty acids
QoL	Quality of life
FODMAP	Fermentable Oligosaccharides, Disaccharides, Monosaccharides, and Polyols
AMY1	Salivary amylase gene 1
GPR41	G protein-coupled receptor 41
GPR43	G protein-coupled receptor 43
RS1–RS5	Types 1 to 5 of resistant starch
RCT	Randomized controlled trial
HDAC	Histone deacetylase
PYY	Peptide YY
GLP-1	Glucagon-like peptide-1
CNS	Central nervous system
SIBO	Small intestinal bacterial overgrowth
BDNF	Brain-derived neurotrophic factor
FMT	Fecal microbiota transplantation
GI	Gastrointestinal
VOC	Volatile organic compound
